# Virulence and antimicrobial resistance factors of *Enterococcus*spp. isolated from fecal samples from piggery farms in Eastern Cape, South Africa

**DOI:** 10.1186/s12866-015-0468-7

**Published:** 2015-07-04

**Authors:** Benson C Iweriebor, Larry C Obi, Anthony I Okoh

**Affiliations:** SA-MRC Microbial Water Quality Monitoring Centre, University of Fort Hare, Alice, 5700 Eastern Cape South Africa; Applied and Environmental Microbiology Research Group, Department of Biochemistry and Microbiology, University of Fort Hare, Alice, 5700 Eastern Cape South Africa; Academic and Research Division, University of Fort Hare, Alice, 5700 Eastern Cape South Africa

**Keywords:** Virulence factors, Multiple antimicrobial resistance, vancomycin resistance, *Enterococcus* spp

## Abstract

**Background:**

Enterococci have emerged as an important opportunistic pathogen causing life-threatening infections in hospitals. The emergence of this pathogen is associated with a remarkable capacity to accumulate resistance to antimicrobials and multidrug-resistance particularly to vancomycin, erythromycin and streptomycin have become a major cause of concern for the infectious diseases community. In this paper, we report the prevalence of *Enterococcus* in respect to species distribution, their virulence and antibiogram profiles.

**Methods:**

Four hundred fecal samples were collected from two piggery farms in the Eastern Cape Province of South Africa. *Enterococcus* species were isolated and confirmed with generic specific primers targeting the *tuf* gene (encoding elongation factor). The confirmed isolates were speciated with enterococci species specific primers that aimed at delineating them into six species that are commonly associated with infections in humans. Antibiotic susceptibility testing was performed by disc diffusion method. Six virulence genes and antimicrobial resistance profiles of the isolates were evaluated molecularly.

**Results:**

Molecular identification of the presumptive isolates confirmed 320 isolates as *Enterococcus* spp. Attempt at speciation of the isolates with primers specific for *E. faecalis, E. durans, E. casseliflavus, E. hirae* and *E. faecium* delineated them as follows: *E. faecalis* (12.5 %), *E. hirae* (31.25 %), *E. durans* (18.75 %) and *E. faecium* (37.5 %) while *E. casseliflavus* was not detected*.* All the isolates were resistant to vancomycin, streptomycin and cloxacillin, and to at least two different classes of antibiotics, with 300 (93.8 %) isolates being resistant to five or more antibiotics. Also, three out of the six virulence genes were detected in majority of the isolates and they are Adhesion of collagen in *E. faecalis* (*ace)* (96.88 %), gelatinase (*gelE)* (93.13 %) and surface protein (*esp)* (67.8 %).

**Conclusion:**

There was high prevalence of multi-resistant vancomycin *Enterococcus* spp. (VREs) in the fecal samples of pigs in the farms studied, and this poses health implications as vancomycin is an important drug in human medicine. Further studies are needed to determine the spread of vancomycin resistance among bacteria of human origin in the communities.

## Background

Generally, antibiotic-resistant pathogens are able to cause a major clinical challenge in both human and animals. *Enterococcus* spp., ubiquitous in nature and a common commensal of the intestinal microbiota of humans and animals, have emerged as one of the most prevalent nosocomial pathogens worldwide [1]. Several factors such as their propensity and inherent ability to acquired resistance to antimicrobials [2], putative virulence traits, biofilm forming capability [3], and ability to horizontally transfer antimicrobial resistance and virulence determinants to other bacteria [4] are attributable to these abilities.

*Enterococcus* spp. are Gram-positive bacteria known to possess a low level of pathogenicity and can cause urinary tract infections, endocarditis, peritonitis, among other diseases. [5, 6]. Until 1988, Vancomycin was one of the preferred antibiotics for the treatment of infections caused by *Enterococcus* spp. But this seems to have changed when the first vancomycin-resistant *Enterococcus* spp. (VREs) were isolated and identified in Great Britain[7]. Also, VREs have been detected and isolated in many countries throughout the world thus heightening the sense of urgency regarding the global presence of antibiotic resistant enterococci[8–10]. As infections caused by VREs are difficult to treat, VRE should be considered a dangerous pathogen as it could easily spread to people with compromised immune system. The risk of death from vancomycin-resistant enterococci (VRE) is has been reported to be about 75 %, compared with 45 % for those infected with a susceptible strain [11]. According to the degree of resistance to vancomycin and teicoplanin, as well as the origin and transferability of the antibiotic resistance genotype, VREs are categorized as expressing the *van*A*, van*B*, or van*C*, van*D*, van*E *and van*G phenotypes [12, 13]. The *van*A phenotype shows a high level of resistance to both vancomycin and teicoplanin, while the *van*B phenotype shows various levels of resistance to vancomycin but is sensitive to teicoplanin. The *van*C phenotype can be further divided into 3 classes, *van*C-1, *van*C-2, and *van*C-3; *van*C-2 and *van*C-3 are expressed as *van*C-2/3 because of their similar genetic sequence [14, 15]. Vancomycin resistance among enterococci spread via the dissemination of mobile genetic elements of variants of the vanA-type element Tn1546 mostly located on conjugative plasmids [16, 17].

A variety of antibiotics are applied at both therapeutic and sub-therapeutic levels in the management of farm animals. Tylosin, a member of the macrolide family is widely used as antimicrobial growth promoters (AGPs). The use of avoparcin has been associated with high level of vancomycin-resistant enterococci in farm animals [18]. The possibility of transmission of bacteria from animals to humans is not limited to zoonotic diseases and the selection of a reservoir of resistant opportunistic human pathogens and possible transmissible resistance determinants through the indiscriminate use of antimicrobials in farm animal managements may have undesirable consequences for human health [18]. The ability of *Enterococcus* spp. to acquire antibiotic resistance through transfer of plasmids and transposons, chromosomal exchange, or mutation presents a significant challenge for therapeutic measures. In addition to this inherent capacity of enterococci to acquire resistance determinants, they possess several virulence factors. The virulence of this organism is associated with several genes such as *ace* (collagen binding cell wall protein), *acm* (surface-exposed antigen), *agg* (aggregative pheromone-inducing adherence to extra-matrix protein), *esp* (enterococcal surface protein), *hyl* (hyaluronidase), *cad1* (pheromone cAD1 precursor lipoprotein), the cAM373 gene (sex pheromone cAM373 precursor), the cCF10 gene (pheromone cCF10 precursor lipoprotein), *cob* (pheromone cOB1 precursor/lipoprotein, YaeC family), *cpd1* (pheromone cPD1 lipoprotein), *cylABLM* (hemolysin), *efaAEfs* (endocarditis-specific antigen), *sagA* (secreted antigen), and *gelE* (gelatinase) [19, 20]. These virulence factors have been reported in enterococci isolated from food of animal origin [21].

The Eastern Cape Province of South Africa is largely rural and agrarian with many commercial piggery farms. The use of antibiotics to manage animal productivity is a common phenomenon and the impact of bacteria with resistance determinant shed into the environment through fecal materials of animal possess a huge epidemiological problem considering the fact that the province has one of the highest HIV/AIDS prevalence in the country. In this paper, we report on the virulence and antimicrobial resistance profiles of *Enterococcus* spp. isolated from fecal samples collected from piggery farms in the Nkonkonbe municipality in the Eastern Cape Province of South Africa as part of our larger study on the reservoirs of antibiotic resistance in the environment.

## Materials and methods

### Ethical clearance

Ethical clearance was obtained from the University of Fort Hare ethics committee prior to sample collection and cooperation was sought from farmers from whose farms samples were collected.

### Study population and sampling

Details on the study population and sampling procedures are as follows. Briefly, samples were collected from two commercial piggery farms in Nkonkonbe municipality within the Amathole Districts of Eastern Cape Province. A total of 400 samples from two farms were collected for the study. Rectal fecal samples were collected from individual breeder pigs using sterile swab sticks and to avoid duplication of sampling, the pigs were sampled while locked in their respective cages. After collection, samples were shipped on ice to the University of Fort Hare Microbiology laboratory for immediate processing. Data on antibiotic type and treatment history were collected with the purpose of describing the study population. Inventory of the antibiotics in the farmers refrigerators were taken during sampling. Sampling was done between June and August, 2014 at an interval of two samplings fortnightly.

### Laboratory detection of *Enterococcus* spp.

The swab sticks were used to inoculate trypticase soya broth and incubated at 37 °C for 18 to 20 h. These were then sub-cultured onto Bile Aesculin Azide agar and incubated at 37 °C for 24 h. Black dew drop colonies were assumed presumptive for *Enterococcus* species. One colony per plate was picked into a sterile trypticase soya broth and further incubated for 18 h at 37 °C for glycerol stock preparation and preservation at–80 °C for future use.

### DNA Extraction

To extract genomic DNA from the previously stored glycerol stocks, isolates were resuscitated in a 5 ml Todd Hewitt broth at 37 °C for 20 h and cells were recovered from 2 ml of the broth in a sterile Eppendorf and centrifugation was done at 5,000 rpm for 10 min. The supernatant was discarded and the cell deposit washed with normal saline and further centrifuged at 5,000 rpm for 3 min. The cell pellet was then re-suspended in a microcentrifuge tube containing rapid lysis buffer:-100 mM NaCl, 10 mM Tris–HCl pH8.3, 1 mM EDTA pH9.0; 1 % Triton X-100, boiled for 15 min followed by centrifugation at 10,000 rpm and supernatant collected and stored at–20 °C for future use.

### Molecular confirmation of the isolates

This preliminary approach stated above was then followed by polymerase chain reaction (PCR) identification analysis with *Enterococcus* genus-specific primers Ent 1 and Ent 2, as previously reported [22] with *E. faecium* ATCC19434 serving as the positive control. The *tuf* gene of the genus *Enterococcu*s was amplified by PCR which was performed in a 25-μl mixture of 5x buffer (supplied with Taq polymerase), 2.5 mmol/l of MgCl_2_, 2.5 u of Taq DNA polymerase, 200 μmol/l of each deoxynucleoside triphosphate, and 10 pmol of each primer Ent1 5′-TACTGACAAACCATTCATGATG-3′ and Ent2 R: 5′-AACTTCGTCACCAACGCGAAC-3′. The PCR mixture was subjected to a 4-min denaturation step at 94 °C, followed by 35 cycles of 60 s at 94 °C, 60 s at 53 °C, and 60 s at 72 °C, and a final elongation step for 5 min at 72 °C. Verification of PCR products were performed in a 2 % agarose gel electrophoresis at 110 V for 45 min, visualized after staining with ethidium bromide in ALLIANCE.4.7 transilluminator and photographed.

### Species identification

A multiplex polymerase chain reaction (PCR) was performed for *Enterococcus* species identification. Amplification of the genes related to the species-specific identification of *E. faecalis, E. faecium, E. hirae, E. durans*, and *E. casseliflavus* were performed as described previously by Jackson [23].

Two PCR master mixes consisting of different primer sets were prepared. Group 1 was *E. durans, E. faecalis,* and *E. casseliflavus*, and group 2 was *E. faecium* and *E. hirae*. The Dream Taq PCR Master Mix (2X) consisting of 4 mM MgCl_2_, 0.4 mM deoxynucleoside triphosphate mix and Taq polymerase enzyme (Thermo Scientific.) and 10pMol of each primer pair was added to constituted the reaction mixture in a PCR tube. Primers used are indicated in (Table 1). PCRs were performed in a final volume of 25 μl consisting of 20 μl of master mix and 5 μl of DNA template. Following an initial denaturation at 95 °C for 4 min, products were amplified in 30 cycles of denaturation at 95 °C for 30 s, annealing at 52 °C (*E.. faecalis, E. durans* and *E. casseliflavus*) or 48 °C (for *E. faecium and E. hirae*) for 1 min, and elongation at 72 °C for 1 min followed by a final extension at 72 °C for 7 min. Five microliters of product was electrophoresed on a 2 % Tris-borate-EDTA agarose gel containing 2 μg of ethidium bromide/ml to verify amplification of the targeted genes at 110 V for 45 min. DNA molecular weight marker 100 bp was used as the standard and photographed under UV light transilluminator (ALLIANCE 4.7) Molecular Imager Gel Doc.

**Table 1:** List of primers and control strains

Strain	Primer Sequence 5'-3'	Product size (bp)	Ref
*E. faecalis* ATCC 19433	FL1 ACTTATGTGACTAACTTAACC	360	23
	FL2TAATGGTGAATCTTGGTTTGG		
*E. durans* ATCC 19432	DU1 CCTACTGATATTAAGACAGCG	295	23
	DU2 TAATCCTAAGATAGGTGTTTG		
*E. casseliflavus* ATCC 25788	CA1 TCCTGAATTAGGTGAAAAAAC	288	23
	CA2 GCTAGTTTACCGTCTTTAACG		
*E. faecium* ATCC19434	GAAAAAACAATAGAAGAATTAT	215	23
	FM2 TGCTTTTTTGAATTCTTCTTTA		
*E. hirae* ATCC 8043	HI1 CTTTCTGATATGGATGCTGTC	187	23
	HI2 TAAATTCTTCCTTAAATGTTG		

### Antibiotic sensitivity testing

The antimicrobial susceptibility of all isolates were assessed according to the Kirby-Bauer disk-diffusion method [24] making use of antibiotic discs (MAST DIAGNOSTICS) which were dispensed by automated disc dispenser on Muller Hinton agar (MHA). The following antibiotic impregnated discs were used: clindamycin (2 μg), imipenem (10 μg), neomycin (30 μg), streptomycin (10 μg), vancomycin (30 μg), penicillin G (10 μg), amoxicillin-clavulanic acid (10 μg), ciprofloxacin (5 μg), cephalothin (30 μg), cloxacillin (5ug), erythromycin (15ug) and amikacin (30ug). In the evaluation of the results, strains displaying intermediate resistance were regarded as resistant. All antibiotic discs contained the CLSI [24] approved concentration. The interpretations of zones of inhibition were carried out according to the [24] performance standards for antimicrobial susceptibility testing guideline.

### Detection of antibiotics resistance genes

Polymerase chain reaction was performed on isolates that were resistant phenotypically to vancomycin for the presence of putative vancomycin resistance (*van*A, *van*B, *van*C1, and *van*C2/3) genes from the previously extracted genomic DNA. PCRs were performed in a BioRad Thermal Cycler (CA, Foster City, USA). The oligonucleotide primers for PCR amplifications were synthesized by Inqaba Biotech (Pretoria, South Africa). Primer sequences for vanA, vanB, vanC1, vanC2/3 genes were those previously described by Nam [7] and the list of the specific primers and their amplification products are shown in Table 2. The reactions were performed as singleplex in a total volume of 25 μl, using 5 μl of cell lysate as DNA template, 10 pMol of each of the eight primers, 12 μl of Dream Taq master PCR mix (Inqaba Biotech, Pretoria, South Africa) and 6 μl of PCR water grade. Amplification conditions were as follows: a first denaturation step of 94 °C for 3 min, 35 cycles of denaturation at 94 °C for 1 min, annealing at 56.5 °C for 1 min, extension at 72 °C for 1 min, followed by an elongation step at 72 °C for 10 min. The PCR products were analyzed on 2 % agarose gel containing 10 μl of ethidium bromide, electrophoresed at 110 V for 45 min and visualized under UV transilluminator (ALLIANCE 4.7) and photographed.

**Table 2:** Oligonucleotide primers used in this study to identify vancomycin resistance genes

Gene(s)	Size (bp)	Primer sequence (5' to 3')	Region	Ref
vanA	314	AF-GCGCGGTCCACTTGTAGATA	105-124	7
		AR-TGAGCAACCCCCAAACAGTA	399-418	
vanB	220	BF-AGACATTCCGGTCGAGGAAC	844-863	7
		BR-GCTGTCAATTAGTGCGGGAA	1044-1063	
VanC-1	402	C1F-ATCCAAGCTATTGACCCGCT	290-309	7
		C1R-TGTGGCAGGATCGTTTTCAT	672-691	
VanC-2/3	582	C2F-CTAGCGCAATCGAAGCACTC	100-119	7
		C2R-GTAGGAGCACTGCGGAACAA	662-681	

The presence of *erm*(B) and *str*A genes that could have been responsible for the observed resistance to erythromycin and streptomycin were examined by using the primers pairs erm(B) (ermBN1: 5′-CGAGTGAAAAAGTACTCAACCA-3′, ermBN2: 5′-CGGTGAATATCCAAGGTACG-3′) and strAF: 5′-ATCTGTCTGGAGCGGATTTG-3′ and strAR:5′-CCAGTTCTCTTCGGCGTTAG-3′ respectively. In each PCR tube, a reaction mixture (25 μl) containing 5 μl bacterial DNA, 12 μl of Dream Taq Master Mix (Thermo Scientific) 10 pMol each of primer and 6ul of water of PCR grade was prepared. The reaction mixture in the tube was subjected to 35 PCR cycles of denaturation at 94 °C (1 min), annealing at 55 °C and 56.5 °C respectively for *erm* (B) and *str*A (1 min), and elongation at 72 °C (1 min) in a BioRad thermal cycler with a final elongation at 72 °C for 7 min. PCR products with specific sizes of the resistance genes were detected by agarose gel electrophoresis at 110 V for 45 min, stained with ethidium bromide and visualized under UV light in a transilluminator (ALLIANCE 4.7).

### Screening for virulence genes in *Enterococcus* spp.

The presence of virulence genes were investigated from the previously extracted genomic DNA for all the confirmed isolates. Specific primers for the following five virulence genes: *ace, efa*A*, cyl*A*, gel*E*, esp* and *hyl*E were used as previously described by [25]. The list of the primers used and their amplification products are reported in Table 3. The reactions were performed in a total volume of 25 μl using 5 μl of DNA, 10 pmol of each primer, 12 μl of PCR Dream Taq Master Mix (Thermo Scientific). PCR conditions for *ace* and *gel*E genes were denaturation at 94 °C for 1 min, annealing at 50 °C for 1 min, extension at 72 °C for 1 min for 35 cycles and final extension at 72 °C for 10 min while those for the amplification of the *efa*A*, esp, cyl*A and *hyl*E genes were as follows: denaturation at 94 °C for 1 min, annealing at 56.5 °C for 1 min, extension at 72 °C for 1 min, for 35 cycles with a final extension at 72 °C for 10 min. The PCR products were analyzed on 2 % agarose gel containing ethidium bromide, electrophoresed at 110 V for 45 min, visualized under UV transilluminator (ALLIANCE 4.7) and photographed.

**Table 3:** Oligonucleotides used in this study to amplify the enterococci virulence genes

Gene	Virulence marker	Oligonucleotide sequence(5' to 3')	Product size (bp)	Annealing temp(C)	References
As	Aggregation substance	1 CCAGTAATCAGTCCAGAAACAACC	406	54	25
		AS 2 TAGCTTTTTTCATTCTTGTGTTTGTT			
Ace	Adhesion colaagen in E. faecalis	ACE 1 AAAGTAGAATTAGATCCACAC	320	56	25
Gel	Gelatinase	gel E1 AGTTCATGTCTATTTTCTTCAC	402	56	25
		gel E2 CTTCATTATTTACACGTTTG			
EfaA	E. faecalis antigen A	efaA1 CGTGAGAAAGAAATGGAGGA	499	56	25
		efaA2 CTACTAACACGTCACGAATG			
Esp	Surface protein	Esp 46 TTACCAAGATGGTTCTGTAGGCAC	913	58	32
		Esp 47 CCAAGTATACTTAGCATCTTTTGG			
CylA	Cytolisin	Cyl I ACTCGGGGATTGATAGGC	688	56	32
		Cyl Iib GCTGCTAAAGCTGCGCTT			
Hyl	Hyaluronidase	Cyl Iib GCTGCTAAAGCTGCGCTT	276	56	
		Hyl n1 ACAGAAGAGCTGCAGGAAATG Hyl n2 GACTGACGTCCAAGTTTCCAA			

## Results

**Fig. 1:**
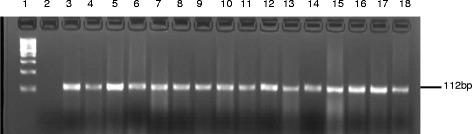
Gel electrophoresis of PCR product of amplification of *tuf* gene for confirmation of Enterococcus genus. Lane 1 is the 100bpMWM, lane 2 is the negative control, 3 is positive control *E. faecium* ATCC19434 while lanes 4 to 18 are amplicons derived from study isolates

**Fig. 2:**
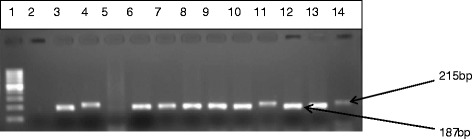
Gel electrophoresis of multiplex PCR product for the speciation of the isolates positive for Enterococcus genus. Lane 1 is 100 bp ladder, lane 2 negative control and lanes 3 to 14 are *E. hirae* (187 bp) and *E. faecium* (215 bp), representatives of the positive isolates identified in this study

**Fig. 3:**
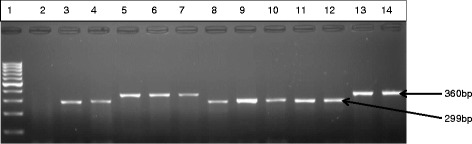
Gel electrophoresis of PCR product multiplex PCR detection of *E. faecalis, E. durans* species isolated in this study. Lane 1 is 100 bp ladder, lane 2 negative control and lanes 3 to 13 are *E. faecalis* (360 bp) and *E. durans* (299 bp), representatives of the positive isolates identified in this study

A total of 320 presumptive isolates were recovered from the 400 fecal samples collected from the breeder pigs in a 600 and 3,000 sizes heard that have been exposed to tylosin, advocin (danofloxacin), ampicillin, and penicillin G antibiotics. Molecular identification of the presumptive isolates based on the *tuf* gene specific primers confirmed them to be *Enterococcus* spp. Representatives of the confirmed isolates are shown in Fig. 1. Attempt at speciation of the isolates with primers specific for *E. faecalis, E. durans, E. casseliflavus, E. hirae* and *E. faecium* delineated them as follow: *E. faecalis* (12.5 %), *E. hirea* (31.25 %), *E. durans* (18.75 %) and *E. faecium* (37.5 %) while *E. casseliflavus* was not detected as shown in Figs. 2 and 3.

### Antibiotic susceptibility

**Fig. 4:**
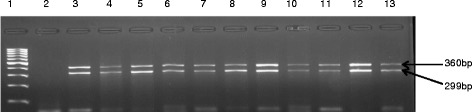
Phenotypic antibiotic resistance of the *Enterococcus* isolates recovered from this study

A very high multi-resistance to antibiotics tested was observed among the isolates. All the isolates were resistant to most of the drugs tested against them with vancomycin, streptomycin and cloxacillin have 100 % resistance respectively. Among the 12 antimicrobial agents tested, the frequencies of resistances to penicillin G (91 %), clindamycin (98.72 %), ciprofloxacin (77.5 %), erythromycin (98.72 %), neomycin (93.8 %), amikacin (85 %), cephalothin (86.3 %) were among the highest while those of imipenem (16.3 %) and amoxicillin/clavulanate (20 %) were least frequent. The phenotypic multi-resistance patterns of the isolates are shown in Table 4 while the percentage resistance is in Fig. 4. All *Enterococcus* isolates were resistant to at least two different classes of antibiotics, with 300 (93.8 %) isolates being resistant to five or more antibiotics. Overall, all the isolates recovered demonstrated relatively high resistance levels to agents that are used in the farms which includes penicillin, erythromycin that is selected by tylosin and quinolones (advocin).

**Table 4:** Antibiotic resistance profiles of *E. faecium, E. hirae, E. durans* and *E. faecalis* isolates obtained from pig faecal samples

Antibiotics	*E.faecium*		*E.hirae*		*E.durans*		*E.faecalis*	
	R	S	R	S	R	S	R	S
VANCOMYCIN	120(100 %)	0(0 %)	100(100 %)	0(0 %)	60(100 %)	0(0 %)	40(100 %)	0(0 %)
CEPHALOTHIN	109(90.8 %)	11(9.2 %)	93(93 %)	7(7 %)	50(83 %)	10(17 %)	34(85 %)	6(15 %)
PENICILLIN G	114(95 %)	6(5 %)	98(98 %)	2(2 %)	45(75 %)	15(25 %)	35(87 %)	5(13 %)
CIPROFLOXACIN	113(94.1 %)	7(5.9 %)	87(87 %)	13(13 %)	30(50 %)	30(50 %)	18(45 %)	22(55 %)
STREPTOMYCIN	120(100)	0(0 %)	100(100 %)	0(0 %)	60(100 %)	0(0 %)	40(100 %)	0(0 %)
AMOXIL/ CLAV	20(16.7 %)	100(83.3 %)	15(15 %)	85(85 %)	15(25 %)	45(75 %)	14(35 %)	26(65 %)
AMIKACIN	117(97.5 %)	3(2.5 %)	95(95 %)	5(5 %)	40(66.7 %)	20(33.3 %)	20(50 %)	20(50 %)
CLINDAMYCIN	116(96.66 %)	4(3.44 %)	100(100 %)	0(0 %)	60(100 %)	0(0 %)	40(100 %)	0(0 %)
ERYTHROMYCIN	118(98.3 %)	2(1.7 %)	100(100 %)	0(0 %)	58(96.7 %)	2(3.3 %)	40(100 %)	0(0 %)
NEOMYCIN	120(100 %)	0(0 %)	98(98 %)	2(2 %)	42(70 %)	18(30 %)	40(100 %)	0(0 %)
IMIPENEM	10(8.3 %)	110(91.7 %)	9(9 %)	91(91 %)	20(33 %)	40(77 %)	13(32.5 %)	27(68.5 %)
CLOXACILLIN	120(100 %)	0(0 %)	100(100 %)	0(0 %)	60(100 %)	0(0 %)	40(100 %)	0(0 %)

### Correlation between antibiotic resistance phenotype and genotype

**Fig. 5:**
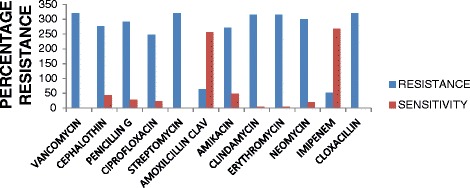
Gel electrophoresis of PCR products of vancomycin resistant isolates. Lane 1 is 100 bp ladder, lane 2 negative control and lanes 3 to 11 are *van*C2/3 (582 bp) positive samples

**Fig. 6:**
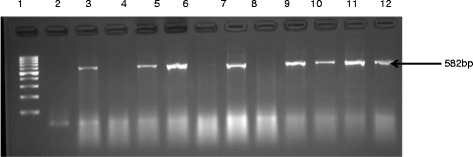
Gel image of amplicons obtained from erythromycin resistant isolates. Lane 1 is 100 bp ladder, lane 2 negative control and lanes 3 to 11 are *erm*B (320 bp) positive samples

Specific resistance genes were detected in corresponding phenotypic antibiotic-resistant isolates (Table 5) and some of the detected resistance genes are shown in Figs. 5 and 6. The detected genes include those conferring resistance to streptomycin an aminoglycosides (*str*A), erythromycin a macrolides (*erm*B), and vancomycin a glycopeptide (*van*B*, van*C1*, van*C2/3). The *van*B*, van*C1*, van*C2/3*, erm*B*,* and *str*A genes were present in majority of the identified species of *Enterococcus* that exhibited phenotypic resistance.

**Table 5:** Predominant multi-resistance pattern observed among the isolates

No. of isolates	Multiple antimicrobial resistance pattern (phenotypic)
320	V/CD/S/E/-/CX/PG/NE/INI/CIP
7	V/CD/S/E/AK/CX/PG/NE/KF/CIP/AUG
2	V/IMI/CD/S/E/-/CX/PG/NE/KF
12	V/CX/E/S/PG/CD/CIP/KF/NE
250	V/CD/S/E/AK/CX/PG/NE/KF

V = Vancomycin, CD = Clindamycin; S = Streptomycin, E = Erythromycin; CIP = Ciprofloxacin; PG = Penicillin G; NE = Neomycin; IMI = Imipenem; KF = Cephalothin; AK = Amikacin; AUG = Amoxicillin/Clavulanic acid

### Genetic prevalence of virulence genes among the isolates

**Fig. 7:**
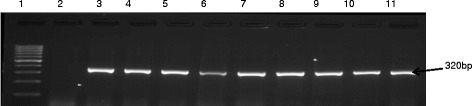
Agarose gel image of amplicons obtained from a multiplex PCR performed with four Primers specific for the *ace* and *gel*E virulent genes of Enterococcus species isolated in this study. Lane 1 is 100 bp ladder, lane 2 negative control and lanes 3 to 13 (*ace* 320 and *gel*E 402) are representatives of the positive isolates identified in this study

Among the virulence genes tested, only *ace, gelE* and *esp* genes were detected in all almost all the isolates that were genetically profiled. The other virulence genes (*cyl*A*, hyl*A*,* and *efa*A) were not detected. The prevalence of the virulence genes detected among the isolates is shown in Table 6. The frequencies of the virulence genes are; *ace* (96.88 %), *gel*E (93.13 %) and *esp* (67.8 %). Fig. 7 represents the gel picture of the *ace* and *gyl*E detected while *esp* was not shown.

**Table 6:** Prevalence of virulent genes amplified from the study isolates

Virulent genes	Number of positive
ace	310(96.88 %)
ge/E	298(93.13 %)
efaA	0
hylE	0
cylA	0
esp	217(67.8 %)

efaA = E.faecalis antigen A; gelE = gelatinase Ace = Adhesion of collagen in E.faecalis

Eesp = surface protein; cylA = cytolysin; hylE = hyaluronidase

## Discussion

*Enterococcus*, which exist commensally in the gut of warm-blooded animals and humans, are opportunistic pathogens that cause a variety of community-acquired and health care–associated infections, such as urinary tract and intra-abdominal infections, bacteremia, and endocarditis [26]. Previous reports have shown that epidemiologically distinct *Enterococcus* spp. possess virulence genes that enable them to establish infections in their host [27] as well as demonstrate high antimicrobial resistance occasioned by their ability to genetically acquire and transmit antimicrobial drug resistant determinants among themselves and other bacteria in their environment [28]. Therefore, we characterized all isolates with respect to these traits. Out of a total of 400 fecal samples collected from breeder pig farms, presumptive isolates recovered from bile aesculine azide medium were 320 and were confirmed as *Enterococcus* spp.by PCR targeting the *tuf* gene of the genus *Enterococcus*. Further characterization of the confirmed 320 isolates by species specific primers delineated them into four species; *E. faecium, E. hirae, E. durans* and *E. faecalis* in order of their prevalence. Our findings are in partial agreement with those of Hwang[29] who reported a preponderance of *E.feacium* and *E.feacalis* in fecal samples of poultry and swine collected at slaughterhouses in South Korea and [30].

The presence of 6 virulence-associated genes (*gel*E, *hyl*A, *ace*, *esp*, *efa*A and *cylA*) was investigated by using gene specific primers that have been described elsewhere [25]. Out of the 6 virulence genes investigated, only three; *ace*, *gel*E and *esp* were detected among the 320 isolates. Our findings are in near agreement with that of Diarra [31] who reported the presence of *gly*E in all isolates they studied in broiler chicken but quite different from that of Mannu [25] who did not detect *gel*E and ace in meat, cheese and vegetable samples that they analyzed. The main role of both gelatinase and serine protease in enterococcal pathogenesis is thought to be in providing nutrients to the bacteria by degrading host tissue and also functions in biofilm formation [32]. Gelatinase (GelE) is an extracellular zinc metallo-endopeptidase secreted by *E. faecalis* [33, 34]. It is able to hydrolyse gelatin, casein, haemoglobin and other bioactive peptides. The gene (gelE) encoding GelE is located on the chromosome and is regulated in a cell-density-dependent manner. Zou [35] reported also a moderately high presence of *efa, gel*E and *ace* virulence genes among *E. faecalis* isolated from swine in China.

The isolates were generally homogenous in terms of presence of virulence-associated genes and it appears from our study that the incidence of known virulence factors in *Enterococcus* is generally high with majority of the strains carrying more than one virulence determinant.

The genes encoding *efa*A*, hyl*A and *cyl*A were however not detected. According to Shankar [32], the *esp* gene encodes an enterococcal surface protein (Esp), which contributes to the colonization and infection of the urinary tract by increasing attachment to epithelial surfaces and biofilm production.

Besides having a huge arsenal of insusceptibilities to physicochemical and environmental stresses [13], *Enterococcus* generally possess a broad spectrum of natural antibiotic resistances [26]. The propensity for multiple antibiotic resistances is a hallmark of *Enterococcus*. All *Enterococcus* are naturally (intrinsically) resistant to many antimicrobial agents such as semisynthetic penicillins (*e.g.*, oxacillin), cephalosporins of all classes, monobactams and polymyxins [37]. Notably, most of the isolates in our study were resistant to the antimicrobials in the panel. Multiple drug resistances were observed among the isolates with the commonest patterns being those of vancomycin, erythromycin and streptomycin. In a study conducted by Diarra [31] on the distribution of antimicrobial resistance of *Enterococcus* spp. in broiler chicken in Canada, they reported a very high level of antimicrobial resistances among their isolates. However, they did not detect vancomycin resistance among their isolates except in few species of *E. gallinarum* where *van*C gene was present making our finds differ a bit from theirs. Similarly, Peters [38] also reported a similar pattern of occurrence of multidrug resistant strains in a study on assessment of species distribution and antibiotic resistance patterns of enterococcal from food of animal origin in Germany. Brown [39] has reported that if glycopeptide resistant enterococci (GRE) are present in an infected patient rather than an antibiotic-susceptible strain, clinical treatment failure is increased by 20 % and mortality is increased from 27 % to 52 %.

The very high level of resistance among the isolates against ciprofloxacin is quite alarming as this is the drug of choice in the treatment of several bacterial infections. It is useful for treating chest, urinary tract, prostatitis, gastroenteritis, bone and joint infections, and some sexually transmitted diseases. Our findings are also in agreement with those of Zou [35] who reported a high level of resistances to erythromycin and ciprofloxacin among *Enterococcus* spp. isolated from swine in China.

The high prevalence of ciprofloxacin resistance could be attributed to the use of advocin a veterinary approved drug that has danofloxacin as it active ingredient. Danofloxacin is a synthetic fluoroquinolone with broad spectrum antibacterial activity and it is commonly used in the treatment of respiratory disease in chickens, cattle and pigs. Since there is similarity in structure and mode of action between ciprofloxacin and danofloxacin, the possibility of cross resistance arising is very high. Genetic investigation of the isolates for the presence of resistance genes yielded amplicons for the *erm*B*, str*A*, van*B and *van*C1/2/3 respectively. The findings in this study are in agreement with previously reported cases of high prevalence of multiple resistances among enterococcal strains of animal origin [40–50]. Noble [51] reported that the genes responsible for vancomycin resistance have the potential to be transferred to other gram-positive pathogens such as *Staphylococcus aureus* thus intensifying the public health threat associated with vancomycin and methicillin-resistant *S. aureus* [52–54, 57–59]. Our findings are also in line with those of Jackson [55] that reported a very high level of aminoglycosides among enterococcal isolated from swine.

The prevalence of antimicrobial resistance among the isolates according to species distribution appears homogeneous as no one species showed disparity with regards to the antimicrobials tested. The high-level resistances among the isolates to neomycin and streptomycin which are the drugs of choice in therapeutic regiments for enterococcal endocarditis should be a major cause of concerns.

Antibiotics may be disseminated into the environment from both human and agricultural sources, including excretion, flushing of old and out-of-date prescriptions, medical waste, discharge from wastewater treatment facilities, leakage from septic systems and agricultural waste-storage structures [56].

Any use of antibiotics will most likely select for drug-resistant bacteria especially when applied at subtherapeutic levels as in animal feed. Among the various uses for antibiotics, low-dose, prolonged courses of antibiotics among food animals have the capacity of creating ideal selective pressures for the evolution and selection of resistant strains. Spread of resistance may occur by direct contact or indirectly through food, water, and animal waste application to farm fields. It could also be augmented greatly by the horizontal transfer of genetic elements such as plasmids via bacterial mating (conjugation). For example, Alexander [60], showed that drug-resistant *Escherichia coli* was present on beef carcasses after evisceration and after 24 h in the chiller and in ground beef stored for 1 to 8 days. Equally ciprofloxacin-resistant *Campylobacter* spp. have been isolated from 10 % to 14 % of consumer chicken products [61, 62] while de Boer [63], have reported the presence of MRSA in 12 % of beef, veal, mutton, pork, turkey, fowl, and game samples purchased in the consumer market in the Netherlands as well as in cattle dairy products in Italy [64]. Equally disturbing are the reports of extensive antibiotic resistance among bacteria isolates, including human pathogens, from farmed fish and market shrimp [65–66].

## Conclusion

The prevalence of multiple antibiotic resistance enterococci from fecal samples of pigs is reported here. The data presented showed that the *Enterococcus* strains that were profiled have the capacity to cause infection as well as having a wide genetic repertoire to survive under antimicrobial pressure. These findings are relevant to public health and contribute to future risk assessment of antimicrobial resistance in zoonotic bacteria. A high-level rate of resistance to aminoglycosides, macrolides and vancomycin might pose a serious risk in hospitals, as antimicrobial therapy in human medicine could become more limited. These findings suggest that *Enterococcus* spp. from swine should be treated with utmost caution as they could be reservoirs for antimicrobial resistance and virulence genes.

## Abbreviations

VREs: vancomycin-resistant *Enterococcus* spp
AGPs: antimicrobial growth promoters
*ace*: collagen binding cell wall protein
*acm*: surface-exposed antigen
*agg*: aggregative pheromone-inducing adherence to extra-matrix protein
*esp*: enterococcal surface protein
*hyl*: hyaluronidase
*gelE*: gelatinase
HIV: human immunodeficiency virus
AIDS: acquired immune deficiency syndrome
PCR: polymerase chain reaction
DNA: deoxyribonucleic acid
